# 2-Hydroxyoleic Acid Induces ER Stress and Autophagy in Various Human Glioma Cell Lines

**DOI:** 10.1371/journal.pone.0048235

**Published:** 2012-10-25

**Authors:** Amaia Marcilla-Etxenike, Maria Laura Martín, Maria Antònia Noguera-Salvà, José Manuel García-Verdugo, Mario Soriano-Navarro, Indranil Dey, Pablo V. Escribá, Xavier Busquets

**Affiliations:** 1 Departmento de Biología - Instituto Universitario de Investigación en Ciencias de la Salud, Universidad de las Islas Baleares, Palma de Mallorca, Spain; 2 Instituto Cavanilles, Universidad de Valencia, Valencia, Spain; 3 Unidad Mixta Centro de Investigación Príncipe Felipe - Universidad de Valencia, Centro de Investigación Biomédica en Red sobre Enfermedades Neurodegenerativas, Valencia, Spain; 4 Lipopharma Inc, Acton, Massachusetts, United States of America; University of Hong Kong, Hong Kong

## Abstract

**Background:**

2-Hydroxyoleic acid is a synthetic fatty acid with potent anti-cancer activity which does not induce undesired side effects. However, the molecular and cellular mechanisms by which this compound selectively kills human glioma cancer cells without killing normal cells is not fully understood. The present study was designed to determine the molecular bases underlying the potency against 1321N1, SF-767 and U118 human glioma cell lines growth without affecting non cancer MRC-5 cells.

**Methodology/Principal Findings:**

The cellular levels of endoplasmic reticulum (ER) stress, unfolded protein response (UPR) and autophagy markers were determined by quantitative RT-PCR and immunoblotting on 1321N1, SF-767 and U118 human glioma cells and non-tumor MRC-5 cells incubated in the presence or absence of 2OHOA or the ER stress/autophagy inducer, palmitate. The cellular response to these agents was evaluated by fluorescence microscopy, electron microscopy and flow cytometry. We have observed that 2OHOA treatments induced augments in the expression of important ER stress/UPR markers, such as phosphorylated eIF2α, IRE1α, CHOP, ATF4 and the spliced form of XBP1 in human glioma cells. Concomitantly, 2OHOA led to the arrest of 1321N1 cells in the G_2_/M phase of the cell cycle, with down-regulation of cyclin B1 and Cdk1/Cdc2 proteins in the three glioma cell lines studied. Finally, 2OHOA induced autophagy in 1321N1, SF-767 and U118 cells, with the appearance of autophagic vesicles and the up-regulation of LC3BI, LC3BII and ATG7 in 1321N1 cells, increases of LC3BI, LC3BII and ATG5 in SF-767 cells and up-regulation of LC3BI and LC3BII in U118 cells. Importantly, 2OHOA failed to induce such changes in non-tumor MRC-5 cells.

**Conclusion/Significance:**

The present results demonstrate that 2OHOA induces ER stress/UPR and autophagy in human glioma (1321N1, SF-767 and U118 cell lines) but not normal (MRC-5) cells, unraveling the molecular bases underlying the efficacy and lack of toxicity of this compound.

## Introduction

2-Hydroxyoleic acid (2OHOA, Minerval), the α-hydroxy derivative of oleic acid, binds to the plasma membrane and alters the organization of its lipids [1], increasing the propensity to form non-lamellar (hexagonal H_II_) lipid phases [1], [2], [3]. Interestingly, this modification inhibits the growth of lung cancer (A549) cells and it induces apoptosis in human leukemia (Jurkat) cells [2], [4], [5]. The changes 2OHOA produces to the structure of the membrane influences the location and activity of amphitropic membrane proteins that are involved in proliferation/differentiation signaling [1], [2], [3], eventually leading to the down-regulation of E2F-1 and dihydrofolate reductase (DHFR), both pivotal proteins in cancer cell proliferation [4], [6]. In this context, although the first steps in the anticancer mechanism of action of 2OHOA are known, the last cellular and molecular events that cause the cancer cell death still remain unclear. In the present study, we provide evidence of the molecular mechanisms underlying the death of various human glioma cell lines, which explains not only the efficacy of this compound against cancer cells but also its safety based on a lack of action against normal cells.

In a cell, the endoplasmic reticulum (ER) fulfills three main functions: 1) protein folding, glycosylation and sorting; 2) synthesis of cholesterol and other lipids; and 3) maintenance of Ca^2+^ homeostasis [7]. Disrupting any of these processes causes ER stress and activates the unfolded protein response (UPR) [7], which can be achieved with a number of cytotoxic agents, such as brefeldin A [8], tunicamycin [9] or the fatty acid palmitate [10]. The molecular elements associated with UPR up-regulate genes that support recovery from ER stress or that initiate apoptosis in cases of severe cell damage [7].

There are three main pathways that mediate UPR signaling: the inositol-requiring enzyme 1 (IRE1) pathway; the eukaryotic translation initiation factor 2a kinase 3 (PERK) pathway; and the activating transcription factor 6 (ATF6) pathway [7]. Key proteins in these pathways include IRE1α (involved in the regulation of apoptosis and the differentiation/proliferation MAPK-dependent pathways) and its ribonuclease product XBP1 (a transcription factor that induces the expression of genes involved in restoring protein folding or degrading unfolded proteins) [11]. Together with XBP1, ATF4 and ATF6 regulate the expression of the C/EBP homologous protein (CHOP), one of the main effectors of ER stress/UPR-induced apoptosis [12]. Another important element is PERK, whose intrinsic kinase activity is induced by oligomerization, resulting in the phosphorylation of the eukaryotic translation initiation factor 2α (eIF2α) and the suppression of global mRNA translation. Under these conditions, only selected mRNAs are translated, including ATF4 [13], which induces the expression of genes involved in the restoration of ER homeostasis and in autophagy [13], [14], [15]. Accordingly, compounds that promote the sustained phosphorylation of eIF2α, such as salubrinal [16], may exert cytoprotective effects. However, prolonged suppression of protein synthesis is incompatible with cell survival, resulting in autophagy [11], and thus eIF2α phosphorylation and ATF4 both stimulate the expression of genes associated with autophagy [13], [14].

Autophagy is a cellular process that mediates the recycling of cytoplasmic macromolecules and structures through the formation of membrane double bounded vacuoles, called autophagosomes, that engulf and degrade large portions of cells [17], [18]. Autophagy has also been associated with the induction of non-apoptotic cell death [11]. The accumulation of misfolded protein aggregates in the ER that cannot be degraded by the proteosome results in the upregulation of the UPR and the expression of autophagy-related genes [14], [19]. Although both the UPR and autophagy can function independently, recent studies have shown that these processes may be linked and share a common function, exerting either cytoprotective (under basal or metabolic stress conditions) or cytocidal effects (after acute cellular damage) [20], [21].

The ER stress is the starting point from which autophagy or apoptosis can be induced. Activation of ER stress and autophagy represents thought a promising therapeutic strategy to treat cancer [22]. As such, we investigated the roles of ER stress and autophagy in the anticancer effects of 2OHOA against human glioma, the most common type of primary tumor in the CNS with one of the highest mortality rates of all cancers [23].

We found that treatment of 1321N1, SF-767 and U118 cells with 2OHOA provoked effects that included: the induction of ER stress-related genes; cell cycle arrest through the accumulation of cells in the G_2_/M phase and autophagic cell death. By contrast, 2OHOA treatment of non-cancer MRC-5 human fibroblast cells failed to induce these key mediators of ER stress, cell growth arrest and autophagy. These findings partly explain the specificity of 2OHOA against glioma cells and the lack of undesired toxic effects when animals are treated with this compound [4]. In addition, this novel therapeutic approach may constitute an innovative treatment for gliomas with very high mortality rates, based on the specific induction of ER stress and autophagy.

## Results

### 2OHOA impairs cell proliferation and viability in 1321N1, SF-767 and U118 human glioma cells

**Figure 1:**
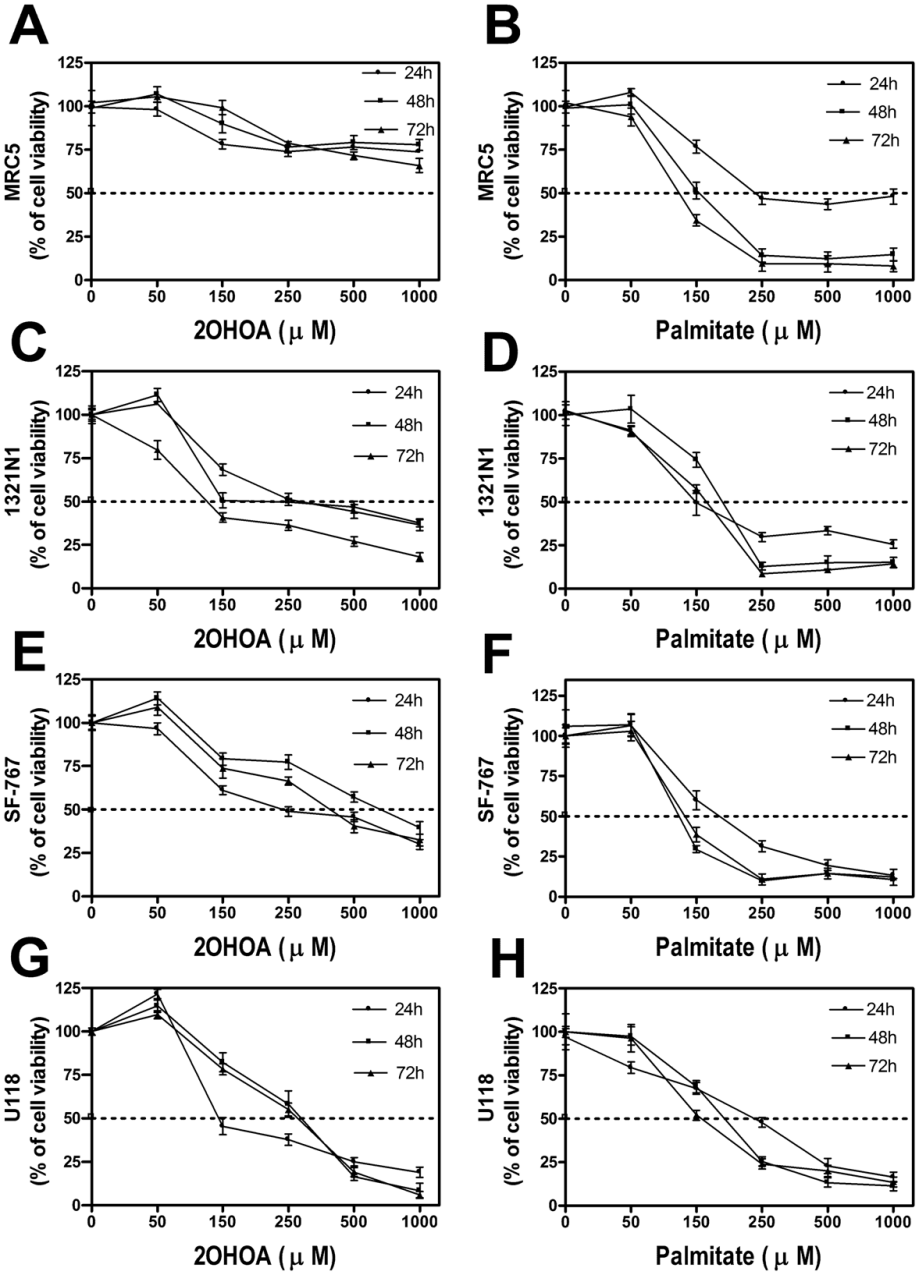
Effects of 2OHOA and palmitate on the proliferation of MRC-5 (A, B), 1321N1 (C, D), SF-767 (E, F) and U118 (G, H) cells. Human glioma (1321N1, SF-767 and U118) cells and fibroblasts (MRC-5) were exposed to increasing doses (50–1000 µM) of 2OHOA or palmitate for different periods of time (24 h, 48 h or 72 h), and cell viability was determined using the MTT method. **A.** Treatments with 2OHOA did not inhibit MRC-5 cell growth below 50% at the highest incubations concentrations and times, so that IC_50_ value could not be determined. **B.** By contrast, the IC_50_ values for palmitate in MRC-5 cells were: 24 h, 200 µM; 48 h, 150 µM and 72 h, 120 µM (n = 6). **C.** The IC_50_ values for 2OHOA in 1321N1 cells were: 24 h, 250 µM; 48 h, 150 µM and 72 h, 100 µM (n = 6). **D.** The IC_50_ values for palmitate in 1321N1 cells were: 24 h, 160 µM; 48 h, 200 µM and 72 h, 160 µM (n = 6). **E.** The IC_50_ values for 2OHOA in SF-767 cells were: 24 h, 600 µM; 48 h, 350 µM and 72 h, 200 µM (n = 6). **F.** The IC_50_ values for palmitate in SF-767 cells were: 24 h, 160 µM; 48 h, 120 µM and 72 h, 110 µM (n = 6). **G.** The IC_50_ values for 2OHOA in U118 cellswere: 24 h, 150 µM; 48 h, 265 µM and 72 h, 260 µM (n = 6). **H.** The IC_50_ values for palmitate in U118 cells were: 24 h, 250 µM; 48 h, 175 µM and 72 h, 150 µM (n = 6).

In order to evaluate cell proliferation in the different cell lines after the treatment with 2OHOA or palmitate, we performed the MTT assay based on the mitochondrial function (succinate dehydrogenase activity). We observed that 2OHOA (50–1000 µM, 24–72 h) had modest effects on the cell proliferation of non-cancer human fibroblast MRC-5 cells (Fig. 1 A), while palmitate, a potent inducer of ER stress that was used as a positive control [10] significantly impaired MRC-5 cell proliferation (Fig.1 B). By contrast, 2OHOA and palmitate, inhibited the proliferation of 1321N1 human astrocytoma cells (Fig. 1 C and D), SF-767 (Fig. 1 E and F) and U118 (Fig. 1 G and H) human glioma cells, demonstrating that only 2OHOA but not palmitate was specific against these glioma cell lines.

**Figure 2:**
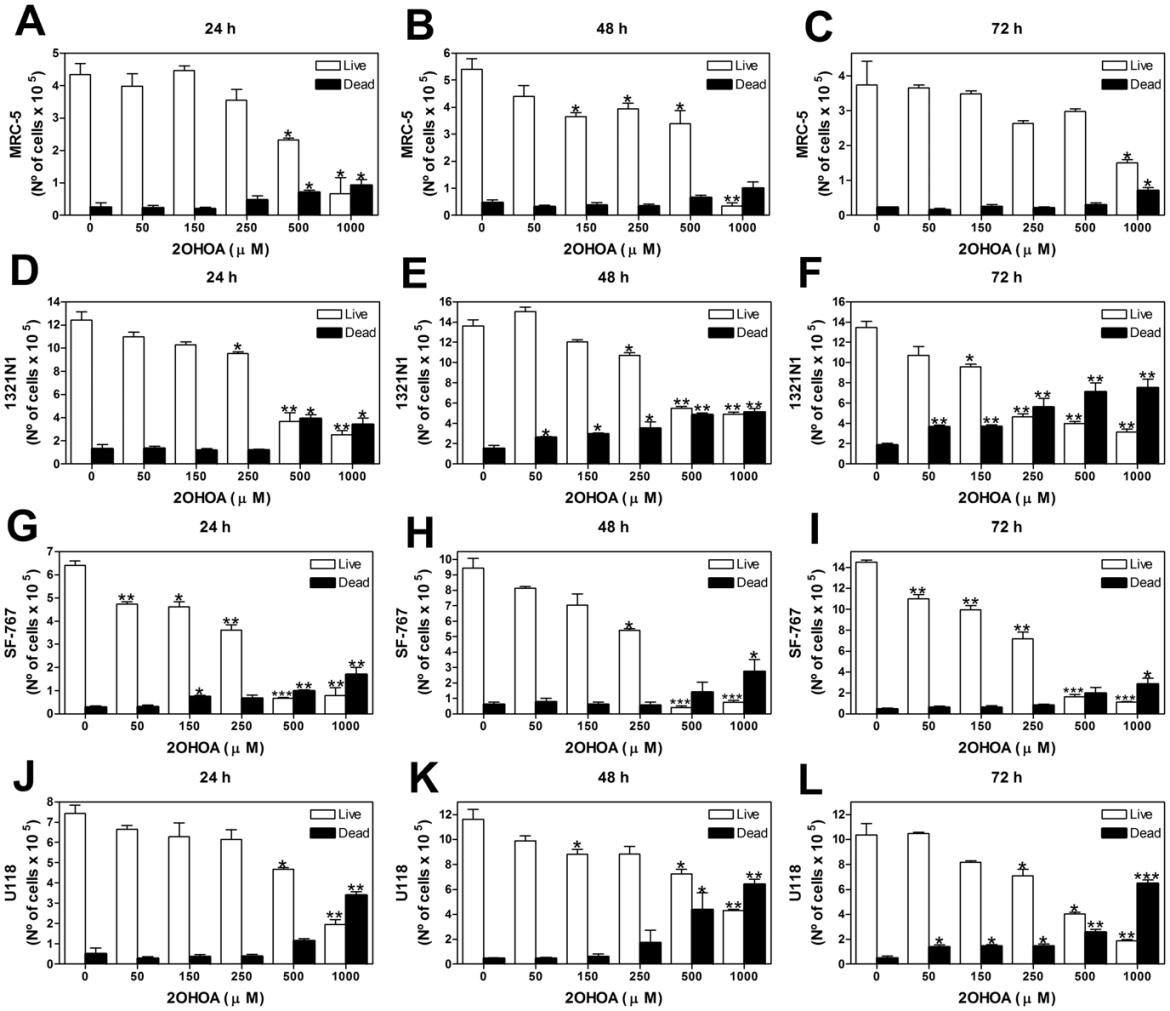
2OHOA effects on cell viability in 1321N1, SF-767 and U118 human glioma cells and MRC-5 human fibroblasts (Trypan blue exclusion method). Glioma and MRC-5 non-tumor cell viability. 1321N1, SF-767 and U118 human glioma cells and MRC-5 human fibroblasts were exposed to increasing doses (50–1000 µM) of 2OHOA for different periods of time (24 h, 48 h or 72 h). Total number of live and dead MRC-5 cells treated with 2OHOA 24 h (**A**), 48 h (**B**), and 72 h (**C**), Total number of live and dead 1321N1 cells treated with 2OHOA 24 h (**D**), 48 h (**E**) and 72 h (**F**). Total number of live and dead SF-767 cells treated with 2OHOA 24 h (**G**), 48 h (**H**) and 72 h (**I**). Total number of live and dead U118 cells treated with 2OHOA 24 h (**J**), 48 h (**K**) and 72 h (**L**). The number of cells presented in the graphs is the total number of cells per well (9.6 cm^2^). Cells were plated at 50% confluence at the following densities: 2×10^4^ cells/cm^2^ (1.86×10^5^ cells/well) for MRC-5 cells; 6×10^4^ cells/cm^2^ (6×10^5^ cells/well) for 1321N1cells and 3×10^4^ cells/cm^2^ (3×10^5^ cells/well) for SF-767 and U118 cells. After 72 h confluence was reached. (*p<0.05, **p<0.01, ***p<0.001; n = 3).

To further analyze cell viability we also used the Trypan Blue Exclusion method, and it was observed that 2OHOA (50–1000 µM, 24–72 h) had modest effects on the cell viability and proliferation of non-cancer human fibroblast MRC-5 cells, with the exception of the highest dose of 1000 µM (Fig. 2 A–C). It cannot be ruled out however, that 2OHOA can also kill MRC-5 non cancer cells in a dose-dependent manner albeit at higher killing concentration. By contrast, 2OHOA (50–1000 µM, 24–72 h) inhibited the proliferation and increased cell death in a time and dose-dependent manner in 1321N1 (Fig. 2 D–F), SF-767 (Fig. 2 G–I) and U118 (Fig. 2 J–L) human glioma cells.

### 2OHOA activates ER stress/UPR signaling pathways in 1321N1, SF-767 and U118 but not MRC-5 cells

**Figure 3:**
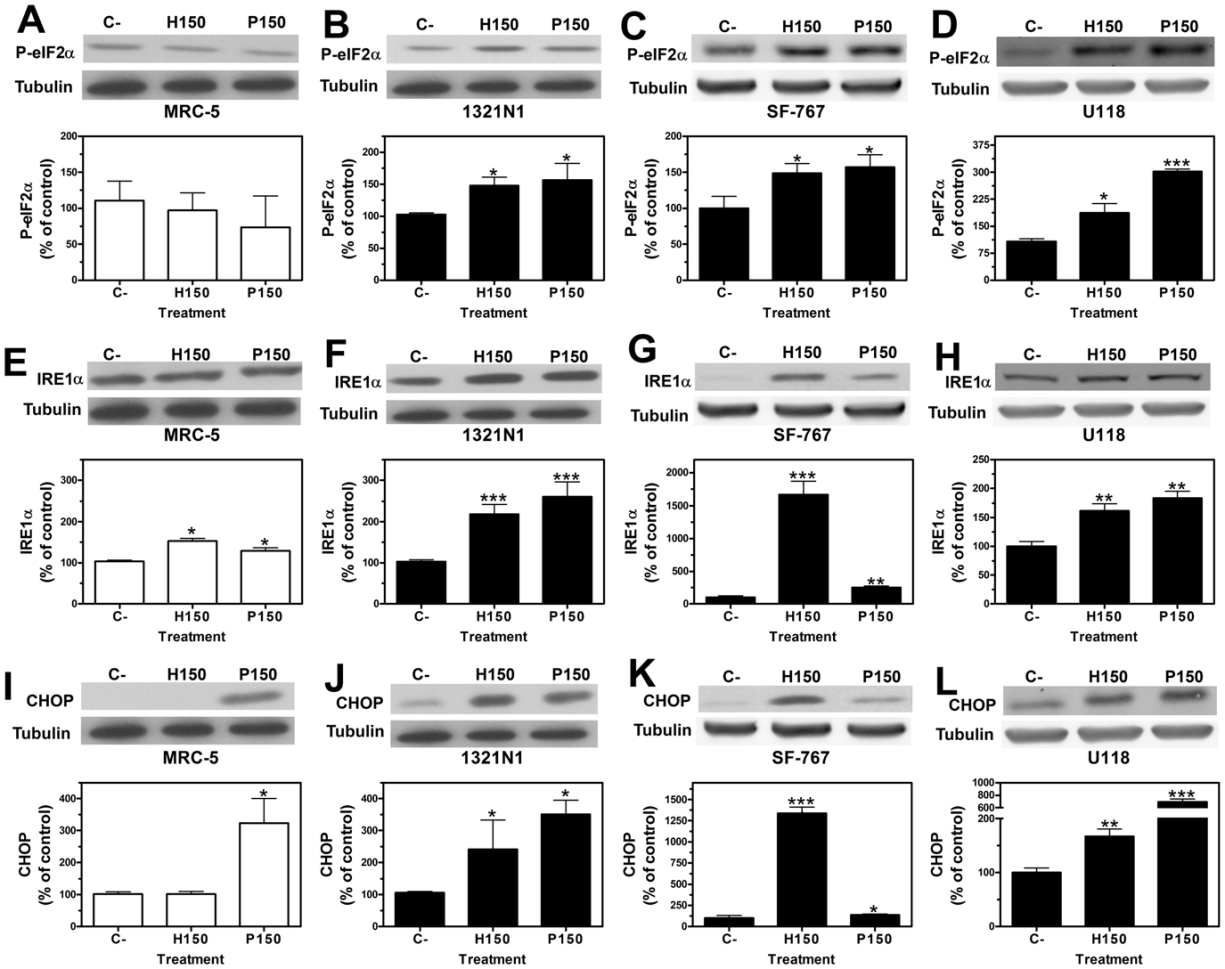
2OHOA activation of ER stress/UPR signaling pathways in 1321N1, SF-767 and U118 but not in MRC-5 cells. P-eIF2α, IRE1α and CHOP protein levels in 1321N1, SF-767 and U118 human glioma cells and in non-cancer MRC-5 human fibroblast cells determined by immunoblotting. Upper panels: a representative immunoblot showing P-eIF2α, IRE1α or CHOP and Tubulin levels in each cell line after exposure to 2OHOA (H) or palmitate (P: 150 µM). Lower panels: Bar diagram showing the mean±SEM P-eIF2α, IRE1α or CHOP expression in each cell line after exposure to 2OHOA (H) or palmitate (P) (150 µM) compared to untreated controls (C). **A.** P-eIF2α expression in MRC-5 cell line **B.** P-eIF2α expression in 1321N1 cell line **C.** P-eIF2α expression in SF-767 cell line **D.** P-eIF2α expression in U118 cell line after exposure to 2OHOA (H) or palmitate (P) (150 µM; 12 h). **E.** IRE1α expression in MRC-5 cell line **F.** IRE1α expression in 1321N1 cell line **G.** IRE1α expression in SF-767 cell line **H.** IRE1α expression in U118 cell line after exposure to 2OHOA (H) or palmitate (P) (150 µM; 48 h). **I.** CHOP expression in MRC-5 cell line **J.** CHOP expression in 1321N1 cell line **K.** CHOP expression in SF-767 cell line **L.** CHOP expression in U118 cell line after exposure to 2OHOA (H) or palmitate (P) (150 µM; 48 h) (*p<0.05, **p<0.01, ***p<0.001; n = 6).

**Figure 4:**
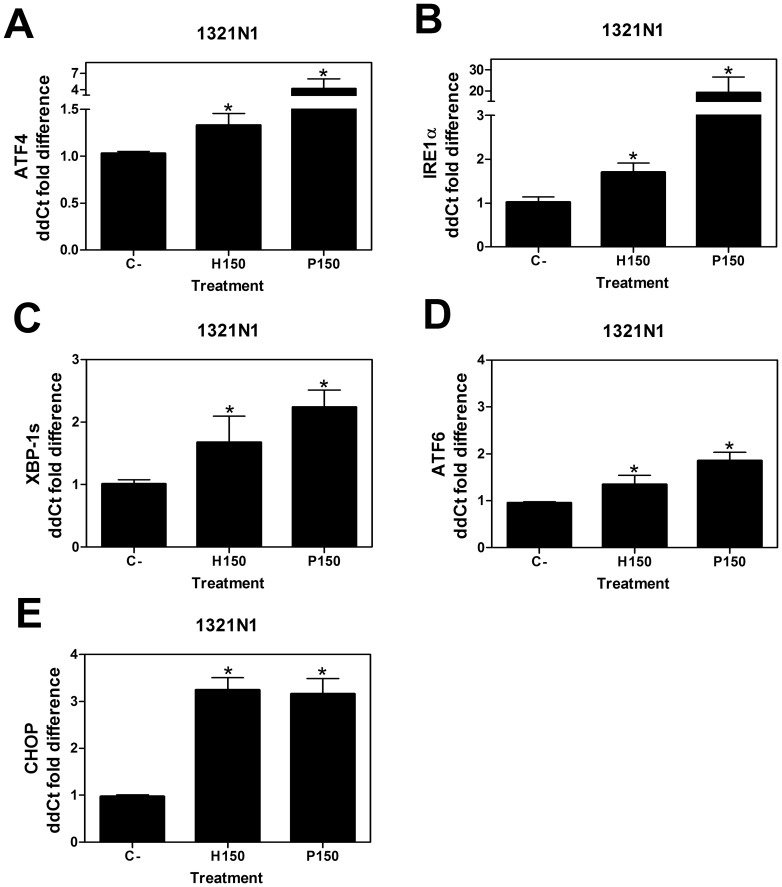
Relative mRNA levels of ER stress/UPR transcripts. q RT-PCR analysis of the mRNA expression of ATF4 (**A**), ; IRE1α (**B**); spliced form of XBP1 (**C**) ; ATF6 (**D**) and CHOP (**E**) genes in 1321N1 human astrocytoma cells after treatment with 2OHOA (H) or palmitate (P) (150 µM; 24 h). Results are expressed as ddCt values using the following formula: ddCt = E X(Ctc-Ctx)/E Bact(Ctc-Ctx). (*P<0.05; n = 6) in a bar diagram showing the mean±SEM (standard error of the mean).

To determine whether inhibition of 1321N1, SF-767 and U118 cell growth by 2OHOA was mediated by ER stress/UPR signaling, we examined the expression of key molecules in the three main signal transduction cascades activated by ER stress/UPR. Treatment of 1321N1, SF-767 and U118 cells with either 2OHOA or palmitate (150 µM; 12 h) significantly increased the P-eIF2α protein levels, while a similar increase in P-eIF2α protein was only produced by palmitate in MRC-5 cells (Fig. 3 A, B, C and D). Thus, the effects of 2OHOA on P-eIF2α accumulation appeared to be specific to glioma cells. Phosphorylated eIF2α attenuates general protein translation and selectively activated transcription and translation of the ATF4 transcription factor [13]. Both 2OHOA and palmitate (150 µM; 24 h) induced a significant increase in *ATF4* gene expression in 1321N1 cells, further demonstrating the specificity of 2OHOA against glioma cells (Fig. 4 A).

Activation of IRE1α resulted in an increase in the expression of the XBP1 transcription factor [24], [25], and 2OHOA and palmitate (150 µM; 24 h and 48 h) markedly up-regulated IRE1α protein levels in 1321N1, SF-767 and U118 cells (Fig. 3 F, G and H) and modestly up-regulated mRNA levels in 1321N1 astrocytoma cells (Fig 4 B). By contrast, the same treatments produced only a mild increase in IRE1α protein expression in MRC-5 cells (Fig. 3 E). The mRNA transcripts of the spliced activated form of the X-box binding protein 1 gene (s*XBP1*), a downstream target of ATF6 and IRE1α augmented in 1321N1 cell line after 2OHOA treatment (150 µM; 24 h) (Fig. 4 C). These observations indicate that 2OHOA activates the UPR signaling in all cell lines, although more weakly in the non-cancerous MRC-5 cells.

We then studied the so-called ATF6 branch of the UPR signaling pathway, which was activated by palmitate (150 µM; 24 h) in 1321N1 cells, provoking a significant up-regulation of *ATF6* mRNA expression (Fig 4 D). In addition, 2OHOA treatment (150 µM; 24 h) also increased significantly *ATF6* mRNA expression in human glioma (1321N1) cells (Fig 4 D).

In situations of chronic ER stress, the P-eIF2α, IRE1α and ATF6 signaling pathways induce the transcription and translation of the proapoptotic factor CHOP. In response to treatment with 2OHOA or palmitate (150 µM) CHOP expression increased in 1321N1, SF-767 and U118 cells, at the protein level (48 h, Fig. 3 J–L) and it also increased at mRNA levels in 1321N1 astrocytoma cells (Fig 4 E). While palmitate administration also increased CHOP protein expression in MRC-5 cells, 2OHOA did not have such effect (Fig. 3 I). Together these findings demonstrate the differential effect of 2OHOA in these glioma cells versus MRC-5 normal human fibroblasts, selectivity not evident with palmitate, which induced ER stress in both normal and glioma cells.

### 2OHOA induces Cell Cycle arrest in 1321N1, SF-767 and U118 but not of MRC-5 cells

**Figure 5:**
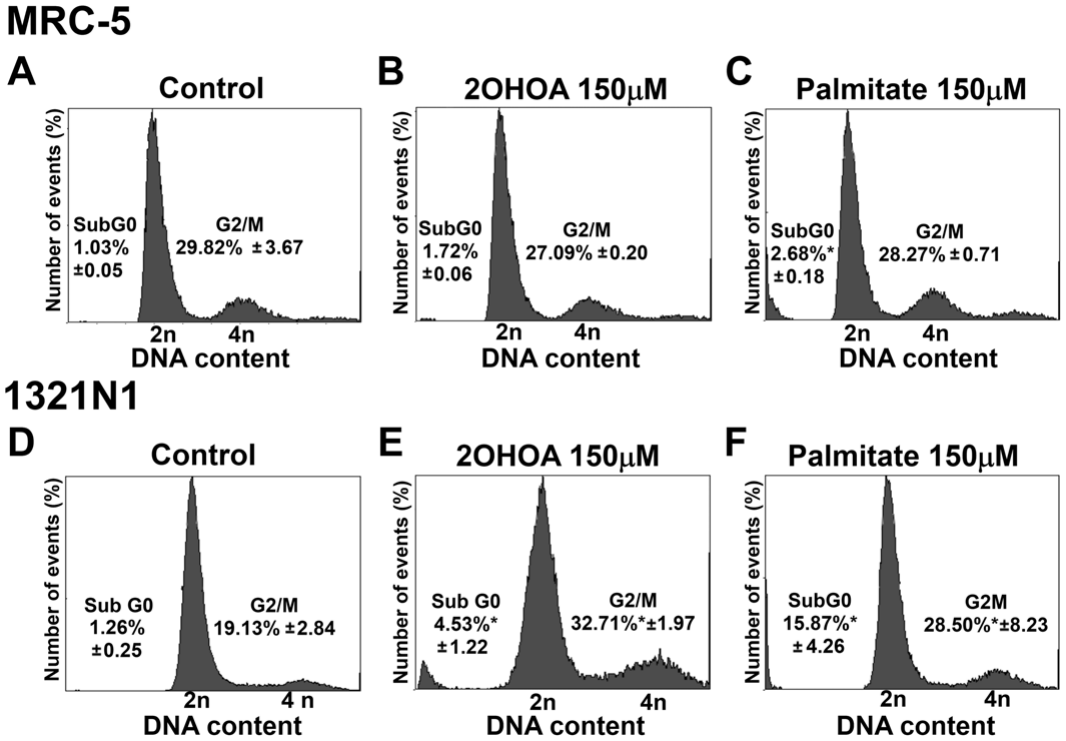
2OHOA induction of G_2_/M cell cycle arrest of 1321N1 cells but not of MRC-5 cells. Cell cycle assessment and G_2_/M phase arrest. Analysis of the DNA content (flow cytometry) of MRC-5 and 1321N1 cells exposed to 2OHOA or palmitate (150 µM for 72 hours). **A.** Analysis of the DNA content in untreated MRC-5 cells. **B.** Analysis of the DNA content in MRC-5 cells exposed to 2OHOA (150 µM for 72 h) or (**C**) palmitate (150 µM for 72 hours), showing the proportion of cells in Sub G_0_ and G_2_/M phases. **D.** Analysis of the DNA content of untreated 1321N1 cells. **E.** Analysis of the DNA content of 1321N1 cells exposed to 2OHOA (150 µM for 72 h) or (**F**) palmitate (150 µM for 72 hours), showing the proportion of cells in Sub G_0_ and G_2_/M phases. Statistical analysis of the DNA content of 1321N1 cells exposed to 2OHOA or palmitate (150 µM) revealed a significant increase (*p<0.05; n = 6) in the G_2_/M phase peak when compared with untreated cells (C-). No significant differences in Sub G_0_ values were detected in MRC-5 cells exposed to 2OHOA (150 µM)) when compared with untreated cells (C-).

**Figure 6:**
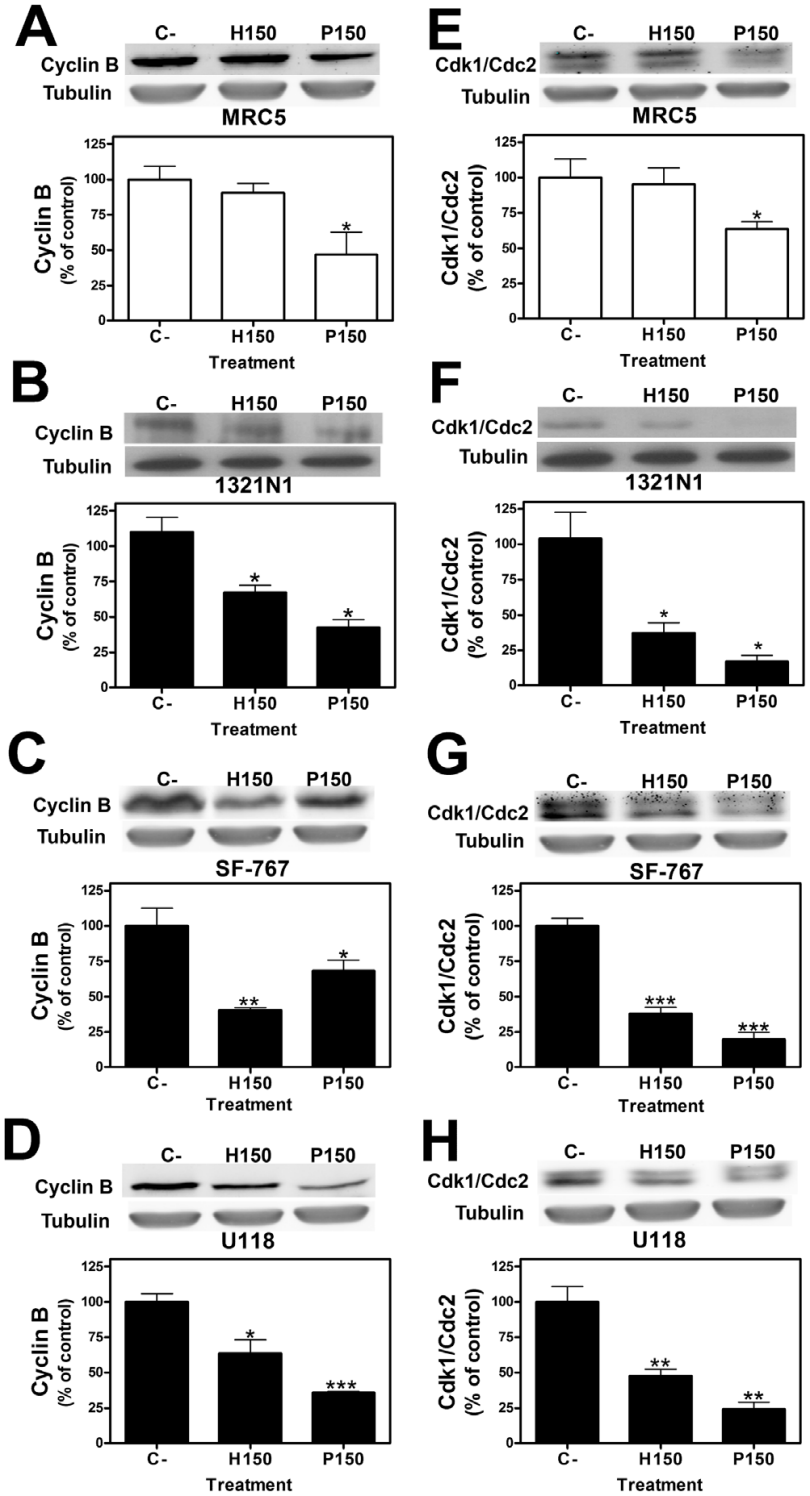
2OHOA inhibition of Cyclin B and Cdk1/Cdc2 proteins in 1321N1, SF-767 and U118 human glioma cells but not in non cancer MRC-5 cells. Cyclin B and Cdk1/Cdc2 proteins in 1321N1, SF-767 and U118 human glioma cells and MRC-5 non cancer cells. A, B, C and D present in the upper panels: a representative immunoblot showing cyclin B expression after exposure to 2OHOA (H) or palmitate (P: 150 µM; 24 hours). Lower panels: Bar diagram showing the mean±SEM values of cyclin B expression in (A) MRC-5, (B) 1321N1, (C) SF-767 and (D) U118 cells, upon exposure to 2OHOA (H) or palmitate (P: 150 µM; 24 h) when compared with untreated controls (C). E, F, G and H show a representative immunoblot of Cdk1/Cdc2 expression after exposure to 2OHOA (H) or palmitate (P: 150 µM; 48 hours, upper panels). The lower panels show the bar diagram showing the mean±SEM values of Cdk1/Cdc2 expression in (E) MRC-5, (F) 1321N1, (G) SF-767 and (H) U118 cells after exposure to 2OHOA (H) or palmitate (P: 150 µM; 48 h) when compared with untreated controls (C). (*p<0.05, **p<0.01, ***p<0.001; n = 6).

The proportion of cells in the different phases of the cell cycle was evaluated by measuring the intracellular DNA content after exposure to 2OHOA and palmitate (150 µM; 72 h). Cell cycle progression and growth of human MRC-5 fibroblast cells was not affected by exposure to 2OHOA (percentage of cells in the G_2_/M phase: Control, 29.82±3.67%; 2OHOA, 27.09±0.20%; palmitate, 28.27±0.71%; *p<0.05. Fig. 5 A–C). By contrast, 2OHOA treatment inhibited 1321N1 cell proliferation, and increased the proportion of cells in the G_2_/M phase when compared to untreated controls (Control, 19.13±2.84%; 2OHOA, 32.71*±1.97%; palmitate, 28.50*±8.23%; *p<0.05. Fig. 5 D–F). Indeed, 2OHOA treatment in 1321N1, SF-767 and U118 induced significant decreases in the expression of both cyclin B1 (Fig. 6 B–D) and Cdk1/Cdc2 (Fig. 6 F–H), indicative of cell cycle arrest in the G_2_/M phase. In contrast, this did not occur in MRC-5 cells (Fig. 6 A and E).

### 2OHOA induces autophagy in 1321N1, SF-767 and U118 but not in MRC-5 cells

**Figure 7:**
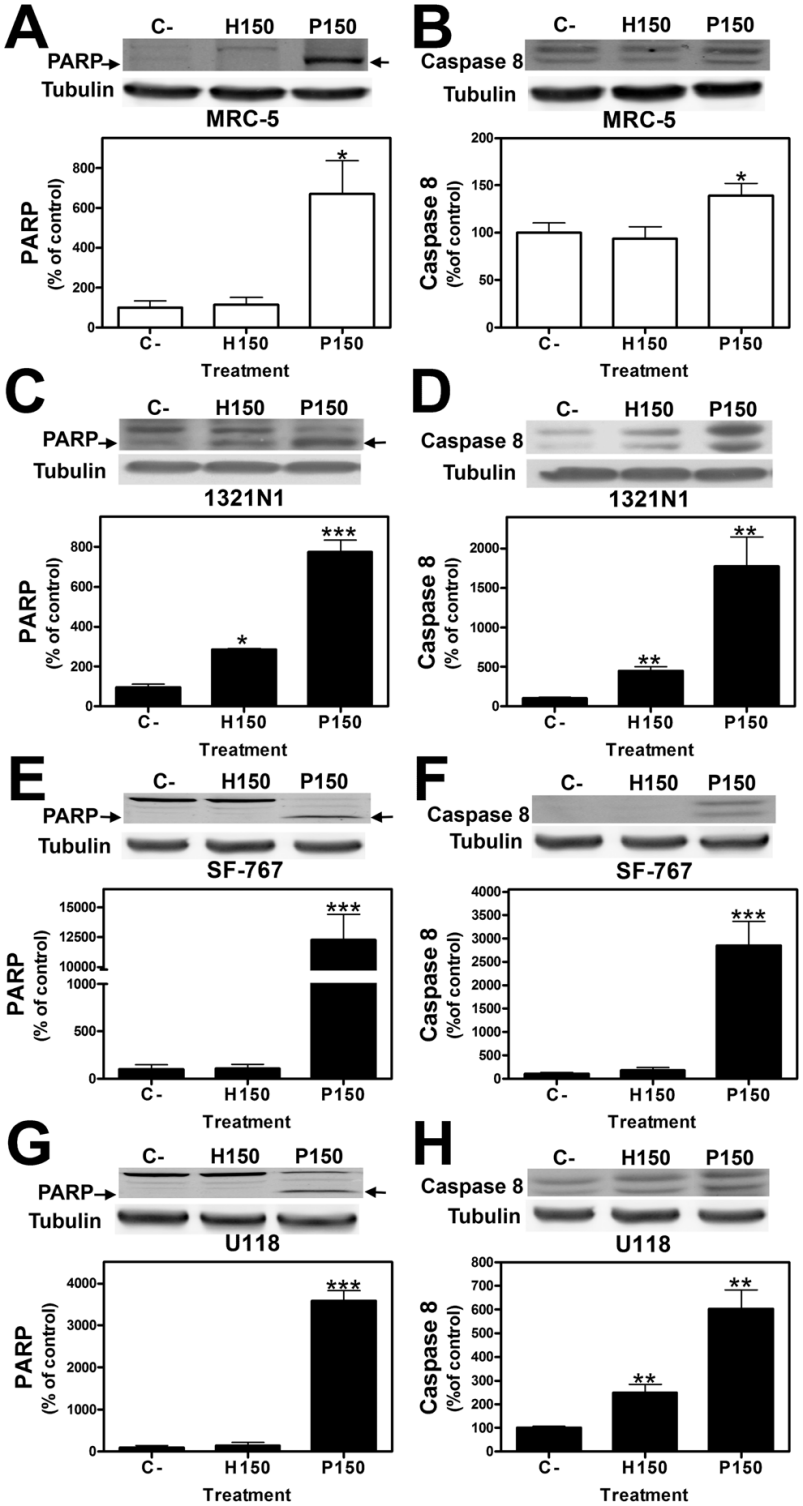
Expression of PARP and Caspase 8 in 1321N1, SF-767, U118 human glioma cells and non cancer MRC-5 cells after treatment with 2OHOA. PARP and Caspase 8 proteins in 1321N1, SF-767, U118 human glioma cells and MRC-5 non cancer cells. Upper panels: a representative immunoblot showing PARP (A, C, E and G) or Caspase 8 (B, D, F and H) expression in every cell line after exposure to 2OHOA (H) or palmitate (P: 150 µM; 72 hours). Lower panels: Bar diagram showing the mean±SEM values of PARP expression in MRC-5 (A), 1321N1 (C), SF-767 (E) and U118 (G) cells or Caspase 8 in MRC-5 (B), 1321N1 (D), SF-767 (F) and U118 (H) cells after exposure to 2OHOA (H) or palmitate (P: 150 µM; 72 h) when compared with untreated controls (C, *p<0.05, **p<0.01, ***p<0.001; n = 6).

**Figure 8:**
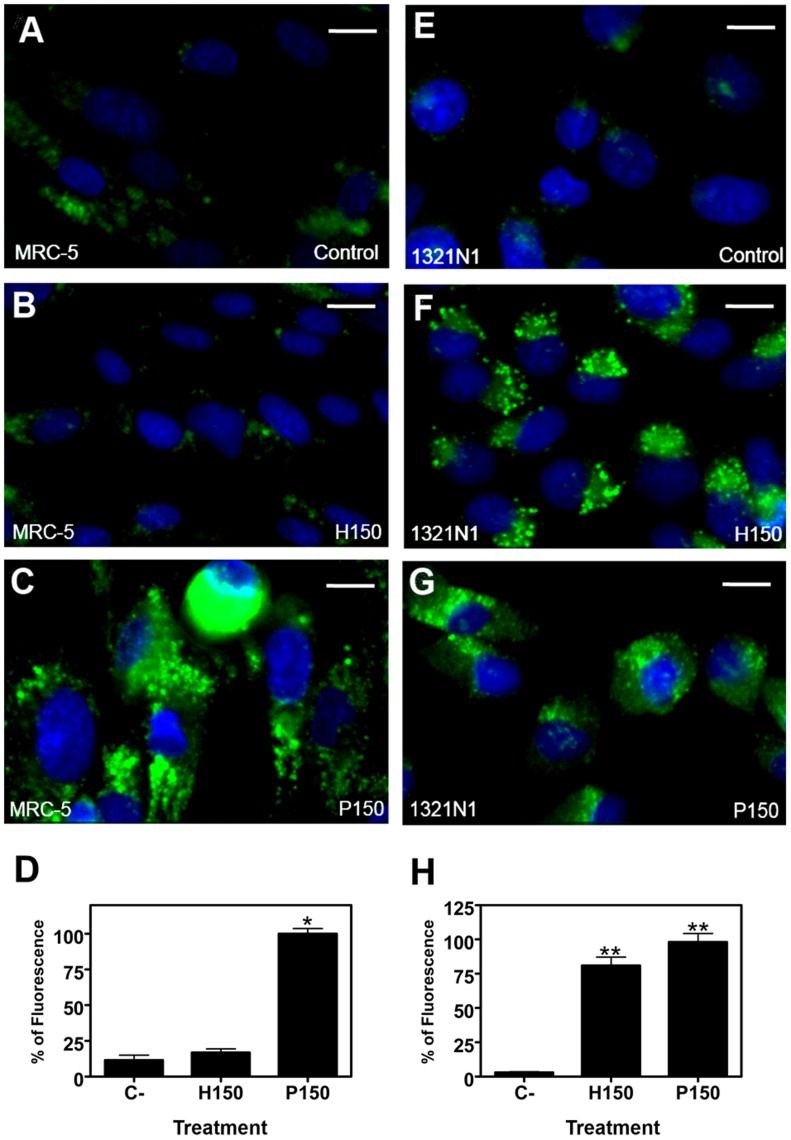
2OHOA induction of acidic vesicles in 1321N1 but not in MRC-5 cells. Analysis of acidic vesicles in cells stained with Hoechst and LysoSensor Green to visualize nuclei and lysosomes, respectively. The images were captured by live cell imaging and they all represent merged images of Hoechst (*blue*) and LysoSensor Green (*green*). The acidic vesicles in photomicrographs were analyzed with Image J 1.38x software. Neither the vehicle (FBS, **A**) nor 2OHOA (150 µM; 48 h, **B**) resulted in the formation of acidic vesicular organelles (lysosomes and autophagosomes) in non-cancer MRC-5 human fibroblast cells, as detected by the LysoSensor fluorescence probe, whereas palmitate (150 µM, 48 h: **C**) induced acidic vesicle formation. Graphs show the integrated fluorescence density of the lysosomes (5×10^4^ cells per experiment) in MRC-5 cells (**D**): Control, 11.54±3.36%; 2OHOA (150 µM), 16.88±2.45%; palmitate (150 µM), 100*±3.65%; *p<0.05. No vesicular organelles accumulated in 1321N1 human astrocytoma cells treated with the vehicle alone (control, **E**), while exposure to 2OHOA (**F**) or palmitate (**G**) (150 µM; 48 h) resulted in the appearance of acidic vesicular organelles. Integrated fluorescence density of lysosomes in 1321N1 cells (5×10^4^ cells per experiment) (**H**): Control, 3.1±0.37%; 2OHOA (150 µM) 81**±6.18%; P (150 µM) 100**±6.12%; **p<0.01 (n = 6 experiments). Scale bar = 10 µm (8A, 8B, 8E, 8F, 8I, 8J); 15 µm (8C, 8G and 8K).

Some features of apoptosis, not observed in MRC-5 cells (Fig.7 A–B), appear to be induced in human astrocytoma (1321N1) cells upon exposure to 2OHOA, such as the flow cytometry sub-G_0_ peak, poly ADP ribose polymerase (PARP) (Fig. 7 C) or caspase 8 partial proteolysis (Fig. 7 D), the latter also observed in U118 cells after treatment with 2OHOA (Fig. 7 H). However, this induction of apoptotic features did not fully explain the cell death induced by 2OHOA in 1321N1, SF-767 and U118 glioma cells as we did not observed PARP degradation induction in SF-767 and U118 cells treated with 2OHOA (Fig. 7 E and G), as well as Caspase 8 proteolysis in SF-767 cells (Fig. 7 E). As 2OHOA induces tumor regression and cancer cell death [6], we also assessed the role of autophagy in the induction of cell death by 2OHOA. Acidic vesicles (lysosomes and autophagosomes characteristic of autophagy) were not observed in non-tumor MRC-5 cells treated with vehicle or 2OHOA (150 µM; 48 h, Figs. 8 A and B), whereas exposure to palmitate (150 µM; 48 h) induced the formation of acidic autophagic vesicles in these cells (Fig. 8 C) The relative integrated fluorescence density of the lysosomes in MRC-5 cells (5×10^4^ cells per experiment) was as follows: Untreated control 11.54±3.36%; 2OHOA (150 µM) 16.88±2.45%; palmitate (150 µM) 100*±3.65%; *p<0.05 (Fig. 8 D).

Both 2OHOA and palmitate (150 µM; 48 h) induced a marked increase in the generation of lysosome/autophagosome vesicles in human astrocytoma (1321N1) cells (Fig. 8 F, G) compared to untreated cells (Fig. 8 E), in which the relative integrated fluorescence density of the lysosomes was: Untreated control 3.1±0.37%; 2OHOA (150 µM) 81**±6.18%; palmitate (150 µM) 100**±6.12%; **p<0.01 (Fig. 8H). Thus, 2OHOA specifically promoted the generation of autophagosomes in cancer cells, whereas palmitate induced unspecific production of acidic vesicles in both normal and cancer cells.

**Figure 9:**
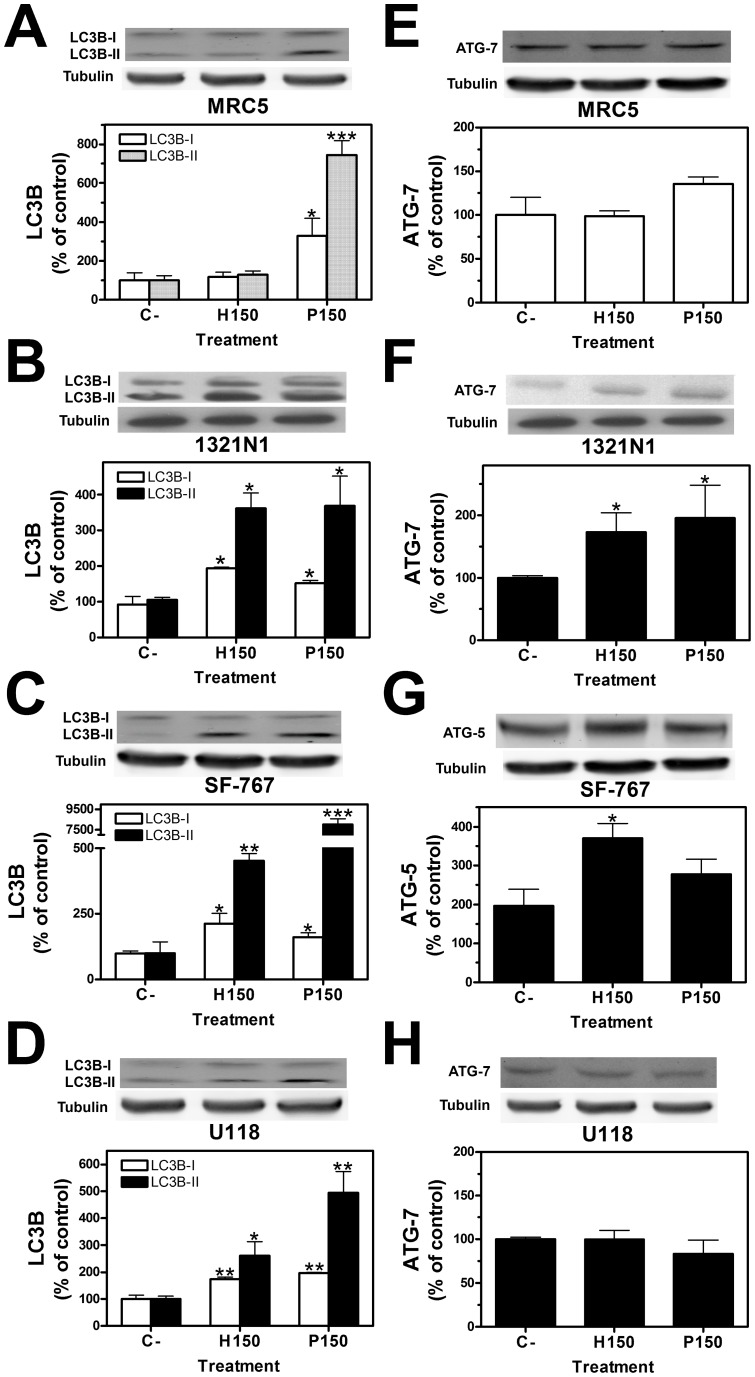
Expression of ATG 7, ATG5 and LC3BI, LC3BII in 1321N1, SF-767, U118 and MRC-5 cells after treatment with 2OHOA. The effects of 2OHOA and palmitate on the levels of ATG 7, ATG5, LC3BI and LC3BII were determined by immunoblots. Exposure of 1321N1 (**B**), SF-767 (**C**) and U118 (**D**) cells to 2OHOA or palmitate (150 µM, 72 h) induced a significant increase in LC3BI and LC3BII protein expression while in MRC-5 (**A**) only palmitate induced significant increases of these proteins. Exposure of 1321N1 cells to 2OHOA or palmitate (150 µM, 72 h) induced a significant increase in ATG7 (**F**) protein expression while in MRC-5 (**E**), and U118 (**H**) did not induce significant changes. Finally exposure of SF-767 cells to 2OHOA or palmitate (150 µM, 72 h) induced a significant increase in ATG5 (**G**) protein expression (*p<0.05, **p<0.01, ***p<0.001; n = 6).

To further confirm that autophagy was induced, we assessed the expression of the autophagy markers ATG7, ATG5, LC3B I and LC3B II. Treatment with 2OHOA or palmitate (150 µM; 72 h) significantly augmented both LC3B-I and LC3B-II in 1321N1, SF-767 and U118 cells compared to MRC-5 cells (Fig. 9 A–D). ATG7 was also up-regulated in 1321N1 cells compared to MRC-5 cells (Fig.9 E and F) and ATG5 was up-regulated in SF-767 cells (Fig.9 G). However, U118 cell line did not show up-regulation of ATG7 (Fig 9 H) nor ATG5 (data not shown) as early as 12 h after treatment, suggesting an earlier induction of this molecules.

**Figure 10:**
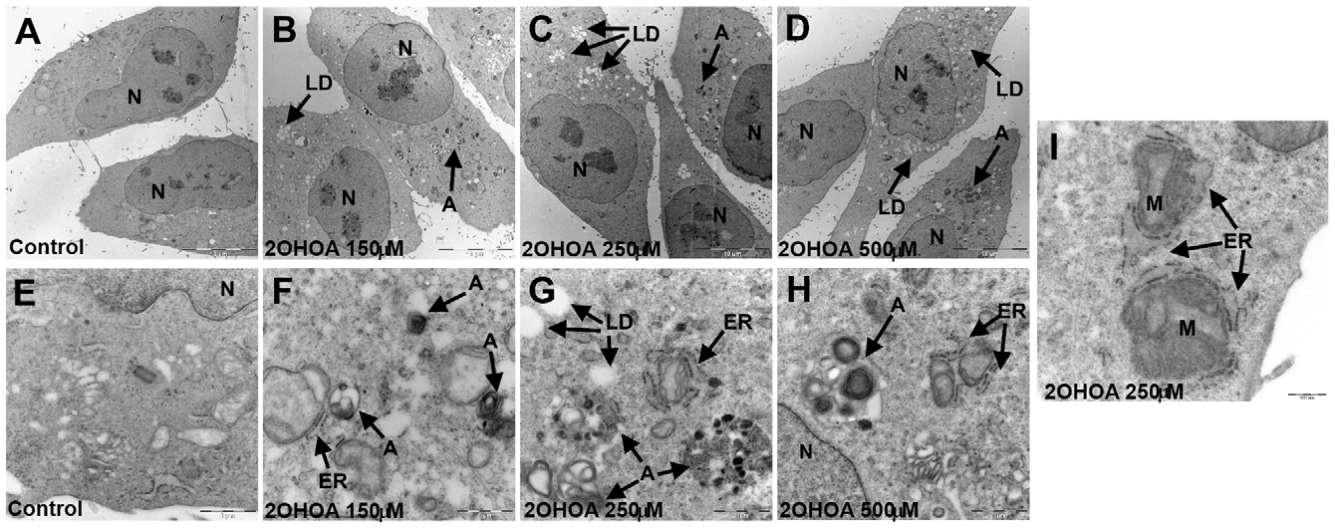
Electron microscopy of 1321N1 cells treated with 2OHOA: induction of Autophagosomes. Electron microscopy of 1321N1 cells maintained for 48 h in the absence (control: 10**A** and 10**E**) or presence of 2OHOA (150 µM: 10**B** and 10**F**; 250 µM: 10**C**, 10**G** and 10**I**; 500 µM: 10**D** and 10**H**). N: Nuclei; A: Autophagosomes; LP: Lipid Droplets; ER: Rough Endoplasmatic Reticulum; M: Mitochondria. Scale bar = 10 µm (10**A**–10**D**); 1 µm (10**E**–10**H**) and 500 nm (10**I**).

Finally, astrocytoma cell degradation upon 2OHOA treatment was further investigated by electron microscopy, which revealed fragments of 1321N1 cells and dense vesicles associated with double layered autophagosomes (Fig. 10). The cytoplasm of control (untreated) 1321N1 cells was densely packed with abundant polyribosomes, mitochondria, dictyosomes and intermediate filament bundles (Fig. 10 A and 10 E). After 48 hours in the presence of the lowest concentration of 2OHOA used in this study (150 µM), the nucleus of 1321N1 cells was no different to that of control cells. Notably, 2OHOA induced the appearance of lipid droplets and dense bodies, the latter scattered throughout the cytoplasm with morphological characteristics of autophagosomes (Fig 10 B to D and 10 F to H). The abundance of these dense bodies was concentration-dependent (Fig 10 B to D), and their heterogeneity increased in function of the concentration of 2OHOA. At both low and high 2OHOA concentrations, distended ER membranes and a loss of ER were observed in the cytoplasm consistent with the ER stress and the autophagic process (Fig.10 F to 10 H). Figure 10I shows in detail early extensions of double endoplasmic reticulum (ER) membranes beginning to surround a mitochondrion, which is characteristic of the autophagic process. These results further support the specificity of the effects of 2OHOA against glioma cells, implicating autophagy as the final cellular effect induced by this compound in these cancer cells.

## Discussion and Conclusions

2OHOA is a potent anticancer drug that inhibits cancer cell growth and induces tumor regression in animal models of cancer, with no undesired side effects. In this context, 2OHOA has been recently granted the status of orphan drug for the treatment of glioma by the European Medicines Agency (EMA). While previous studies have demonstrated 2OHOA provoked cell cycle arrest [2], [4] in cancer cells, the precise molecular and cellular mechanisms underlying the selective induction of glioma cell death is not fully understood.

We investigated the mechanism of 2OHOA-induced cell death in 1321N1 glioma cells for a number of reasons. Firstly, previous studies in our laboratory have demonstrated 2OHOA-induced glioma regression in both animal xenograft models of human glioma and in nude mice (see below). Secondly, unlike most chemotherapeutic agents, this drug is highly selective and it does not induce the death of healthy cells, even at very high doses/concentrations. Finally, while apoptosis has been implicated in the general mechanism of action of 2OHOA against various types of cancer cells [5], SF-767 glioma cells do not initiate the apoptosis program although other lines of glioma cells seem to undergo ER stress and apoptosis [26], [27] and thus, how cell death occurs in such cases remains unknown.

2OHOA selectively inhibits glioma cells growth with an IC_50_ of ∼100 µM in 1321N1, SF-767 and U118 cells as opposed to that of >1000 µM in MRC-5 non cancer cells, which justifies the lack of toxic effects at therapeutic doses. In addition, 2OHOA induces cell cycle arrest in 1321N1, SF-767 and U118 cells, resulting in a significant accumulation of 1321N1 cells in the G_2_/M phase. Indeed, cyclin B and cdk1/cdc2 are downregulated when glioma cells are exposed to 2OHOA. Previous studies have shown glioma cells to undergo autophagy when exposed to compounds that induce cell cycle arrest in the G_2_/M phase [27], [28]. While autophagy provides a means of recycling cytosolic molecules/structures involved in cell survival, it can also represent a non-apoptotic cell death program. Autophagy involves the fragmentation of cells after the engulfment of proteins, organelles and cytosol in vesicles called autophagosomes, which eventually fuse with lysosomes to form autolysosomes [29]. In a variety of cells and tumors, including human glioma, autophagy signaling, the UPR and abnormal cell growth are intimately related [11], [14], [15]. The high rate of cancer cell proliferation is associated with increased protein and lipid synthesis, and active metabolism, which in turn induces a certain level of ER stress [22], [30]. Furthermore, as tumors progresses, cancer cells experience increasing nutrient starvation and hypoxic conditions, resulting in the accumulation of unfolded or misfolded proteins, in turn leading to activation of UPR signaling [11], [22], [30].

Autophagy is triggered in certain situations of stress, with the aim of promoting cell survival by inducing cellular adaptations to the associated conditions [29], [31], [32]. However, increasing evidence suggests that autophagy also serves as a trigger for cell death [29], [31], [32].

As was shown above, some features of apoptosis were induced in 1321N1 and U118 cells but not in SF-767 by exposure to 2OHOA (sub-G_0_ peak, poly ADP ribose polymerase [PARP] or caspase 8 partial proteolysis) (Fig.7). However, this induction of apoptotic features did not fully explain the cell death induced by 2OHOA. Therefore we examined the role of the autophagy induced by the ER stress/UPR signaling pathway in relation to the growth inhibition effects of 2OHOA in 1321N1, SF-767 and U118 human glioma cells and non-cancer MRC-5 cells. Treatment with 2OHOA or palmitate activated ER stress in 1321N1, SF-767 and U118 cells within 12 h, as evidenced by the increase in phosphorylated eIF2α protein, a marker of ER stress. Phosphorylation of eIF2α induces cellular adaptation to various stress conditions by inhibiting protein synthesis and subsequently, by activating expression of the *ATF4* transcription factor [13]. We found that both 2OHOA and palmitate significantly increase *ATF4* expression in 1321N1 cells, while neither eIF2α phosphorylation nor *ATF4* gene expression were evident in non cancer MRC-5 cells exposed to 2OHOA. Along with previous findings, this further demonstrates the specificity of 2OHOA to this glioma cells, explaining the observed lack of side-effects in animal models of cancer.

Since compounds that induce sustained eIF2α phosphorylation provide cytoprotection in situations of ER stress [16], the maintenance of eIF2α in an inactive state is somehow beneficial. However, prolonged suppression of protein synthesis is incompatible with cell survival and leads to autophagy [11], [14], [15]. Exposure of MRC-5 fibroblasts to 2OHOA does not induce eIF2α and *ATF4* expression, or inhibit cell growth, further evidence of its specificity in these glioma cells and demonstrating the role of eIF2α and *ATF4* in 2OHOA-induced cell death of 1321N1, SF-767 and U118 cells.

The second ER stress pathway studied, the IRE1α signaling pathway, was also activated by 2OHOA in 1321N1, SF-767 and U118 cells. 2OHOA induces a significant increase in IRE1α in 1321N1, SF-767 and U118 cells when compared to the modest increase in MRC-5 cells. Interestingly, the spliced activated form of *XBP1s*, a downstream target of ATF6 and IRE1α, was up-regulated by 2OHOA in both 1321N1 and MRC-5 cells. Strong expression of the spliced form of XBP1 is associated with cell survival, whereas expression of the unspliced variant of XBP1 is associated with apoptosis [33]. Our results suggest that the up-regulation of *XBP1* is not essential for cell death, given that it was also observed in MRC-5 cells, suggesting that the activation of other factors besides *XBP1* is necessary to induce autophagy.

Under persistent ER stress, the PERK, IRE1α and ATF6 signaling pathways induce the expression of the pro-apoptotic factor CHOP. In line with its activation of ER stress/UPR, 2OHOA induces CHOP expression in 1321N1, SF-767 and U118 human glioma cells but not in MRC-5 cells, whereas palmitate up-regulated CHOP in glioma and non-cancer cells. As CHOP is one of the most important downstream effector proteins of ER stress, its specific activation by 2OHOA in 1321N1, SF-767 and U118 cells is consistent with the severe induction of ER stress. CHOP activation often leads to the induction of cell death and although CHOP is one of the main effectors of apoptosis [12], 2OHOA did not trigger apoptosis in SF-767 cells despite inducing marked CHOP expression. Nevertheless, activation of autophagy in various glioma cell lines that are usually resistant to apoptosis has recently been associated with CHOP overexpression [34].

We have worked with three human glioma cell lines (1321N1, SF-767, and U118) and as a non-cancer control we have used a human fetal lung fibroblast-like cell line (MRC-5). Despite the differences among the cell lines employed in this study (developmental age and tissue type), a number of mounting evidence suggest that the changes observed here are not due to the developmental age or tissue type. Thus, the efficacy of 2OHOA against different cancer cell types apart from glioma has been reported elsewhere [4], [5], [6], [35]. Moreover, the lack of effects against nomal (non-tumor, IMR-90) cells *in vitro* (cell culture) [5] or *in vivo* (animal models, unpublished GLP toxicology data in mice, rats, and dogs) and the efficacy observed in animal models of glioma [36], supports the specificity of 2OHOA against glioma cancer cells.

Increasing our understanding of the molecular basis of cell death induced by activating ER stress/UPR signaling is of considerable interest, since many proteins in these pathways constitute important potential drug targets [26]. In a previous study [35], we showed that cancer cells have very low membrane sphingomyelin and high phosphatidylethanolamine levels. In glioma and other types of cancer cells but not normal cells, 2OHOA induces changes in these lipids to reach values found in healthy tissues. The present study sheds light on the signaling events that follow the activation of this molecular switch. Here, we demonstrate the selective induction of several key effectors of ER stress/UPR cell death (P-eIF2α, ATF4 and CHOP) by 2OHOA in three human glioma cells. Moreover, we provide cellular and molecular evidence that 2OHOA induces autophagy in these cells, which may constitute a novel therapeutic strategy to combat glioma, when the cells are reluctant to enter apoptosis. As a matter of fact, we have demonstrated that 2OHOA has greater efficacy than the reference drug for the treatment of glioma, temozolomide, in subcutaneous and orthotopic xenograft models of human glioma in nude mice [36]. In conclusion, the design of new lipid molecules like 2OHOA that can modulate ER stress/UPR, constitutes a promising and novel approach to treat gliomas and other neoplasias.

## Materials and Methods

### Cell Culture

Human glial cells from 1321N1 brain astrocytoma, U118 glioblastoma and human fetal lung fibroblast-like MRC-5 cells were obtained from the European Collection of Cell Cultures and SF-767 cells were obtained from the Brain Tumor Research Center Tissue Bank (University of California-San Francisco, Department of Neurological Surgery). They were cultured in Dulbecco's Modified Eagle Medium (DMEM) low glucose medium, supplemented with L-glutamine (2 mM), Non Essential Amino Acids (NEAA, 1%), Fetal Bovine Serum (FBS, 10%), penicillin (100 U/ml) and streptomycin (0.1 µg/ml), at 37°C in a humidified atmosphere of 5% CO_2_. The cell culture medium and supplements were all purchased from Sigma-Aldrich (Madrid, Spain).

### 2-Hydroxyoleic Acid (2OHOA)

2OHOA was obtained from Lipopharma and its purity (99.7%) was confirmed by HPLC and gas chromatography.

### Cell proliferation (MTT) assay

Cell proliferation was determined using the MTT (methylthiazolyl diphenyl tetrazolium bromide) method [37]. 1321N1, SF-767, U118 and MRC-5 cells were plated in 96-well plates at densities of 3×10^3^ cells/well (glioma cells) and 6×10^3^ cells/well (MRC-5), respectively, and with 150 µl culture medium (5% FBS) per well. After incubating overnight to allow cell attachment, the cells were treated with 50–1000 µM of 2OHOA or palmitate for 24 h, 48 h or 72 h, and 10% of MTT (5 mg/ml in PBS 1X) reagent was then added for 4 h. The medium was removed, 200 µl of DMSO was added to the cells for 5 min and they were gently shaken. Absorbance at 550 nm was measured using a Micro Plate Reader.

### Cell viability assay (Trypan Blue exclusion)

Cell viability was determined using the Trypan blue staining method [38]. 1321N1, U118, SF-767 and MRC-5 cells were plated in 6-well plates at densities of 2×10^4^ cells/cm^2^ (1.86×10^5^ cells/well) for MRC-5 cells; 6×10^4^ cells/cm^2^ (6×10^5^ cells/well) for 1321N1 cells and 3×10^4^ cells/cm^2^ (3×10^5^ cells/well) for SF-767 and U118 cells. Cells were plated at 50% confluence and cultured with 2 ml of culture medium (5% FBS) per well. After incubating overnight to allow cell attachment, the cells were treated with 50–1000 µM of 2OHOA or palmitate for 24 h, 48 h or 72 h. After 48 h confluence was reached.

Trypan blue staining was done as previously described [38]. Briefly, 10 µl of sample (cell suspension) was mixed with 10 µl of trypan blue (Invitrgen), and pipeted into Countess® chamber slide (Invitrogen) that was inserted in the Countess® Automated Cell Counter (Invitrogen).

### Electrophoresis, immunobloting and protein quantification

1321N1, SF-767, U118 were plated at densities of 1×10^4^ cells/cm^2^ and MRC-5 cells at 3×10^4^ cells/cm^2^, respectively, in 10 cm^2^ plates containing 8 ml of culture medium (5% FBS). After incubation overnight, the cells were treated with 150 µM of 2OHOA or palmitate for 12 h, 24 h, 48 h and 72 h. Although 2OHOA was diluted in FBS (50 mM) and palmitate in DMSO (100 mM), DMSO was always present at a final concentration of 0.1–1%. After incubating in the presence or absence of 2OHOA or palmitate at the indicated concentrations and times (see the Results section), the cells were washed twice with PBS and harvested with a rubber policeman in 300 µl of protein extraction buffer (10 mM Tris-HCl [pH 7.4], containing 50 mM NaCl, 1 mM MgCl_2_, 2 mM EDTA, 1% SDS, 5 mM iodoacetamide, 1 mM PMSF, 2% cantaridin and 0.1% sodium orthovanadate). Cell suspensions were twice subjected to ultrasonication for 10 s at 50 W using a Braun Labsonic U sonicator and 30 µl aliquots were removed for protein quantification using the BCA method (bicinchoninic acid) [39] (*Pierce - Thermo Fisher Scientific Inc*, Roskilde, Denmark). The remaining suspension (about 270 µl) was mixed with 30 µl of 10X electrophoresis loading buffer (120 mM Tris-HCl [pH 6.8], containing 4% SDS, 50% glycerol, 0.1% bromophenol blue, 10% mercaptoethanol) and boiled for 5 mins. Proteins were fractionated on 8% polyacrylamide gels (SDS-PAGE: 15-well and 1.5 mm thick) and transferred to nitrocellulose membranes (Whatman® protran®, Dassel, Germany). The nitrocellulose membranes were then blocked for 1 h at room temperature in Tris-buffered saline (TBS 1X) containing 5% non-fat dry milk and 0.1% Tween 20 (blocking solution), and the membranes were incubated overnight at 4°C with one of the following primary anti-human antibodies diluted in TBS containing 0.5% bovine serum albumin and 0.1% Tween 20: monoclonal anti-IRE1α, anti-CHOP, anti-P-eIF2α, anti-caspase 8, anti-ATG7, ATG5 and anti-LC3B (1∶1,000, Cell Signaling Technology Inc., Beverly, MA) or polyclonal anti-Cyclin B, anti-Cdk1/Cdc2 (1∶1,000, BD Transduction Laboratories™ Heidelberg, Germany) and anti-PARP (1∶2,000, Sant Cruz Biotechnology, Santa Cruz, CA). After removing the primary antibody, the membranes were washed three times for 10 min with 1X TBS and incubated for 1 h at room temperature in fresh blocking solution with a horseradish peroxidase-linked goat anti-mouse IgG antibody (against monoclonal primary antibodies, 1∶2,000; Amersham Pharmacia) or a horseradish peroxidase-linked goat anti-rabbit IgG antibody (against polyclonal primary antisera, 1∶2,000; Cell Signaling Technology Inc., Beverly, MA). Immunoreactivity was detected using the Enhanced Chemiluminescence Western Blot Detection system (ECL; Amersham Pharmacia) and by exposure to ECL hyperfilm (Amersham Pharmacia). The films were scanned at a resolution of 600 dpi for quantification using the Foto Look 32 software (Agfa Gevaert, Leverkusen, Germany) and the images were analyzed with TotalLab v2005 (*Nonlinear Dynamics*, All Saints, UK) to obtain the integrated optical density (IOD) of each band. The α-tubulin content of each sample was determined by the same procedure and the concentration of a given protein was normalized to the α-tubulin content of the same sample.

### Cell DNA content

To determine cell growth and the cell cycle phase of the cells, the cellular DNA content was determined by staining cells with ethidium bromide followed by single-cell fluorescence flow cytometry. 1321N1 and MRC-5 cells were seeded in 6-well plates containing 2 ml of culture medium (5% FBS) per well at densities of 1×10^4^ cells/cm^2^ and 3×10^4^ cells/cm^2^, respectively, and they were incubated for 72 h in the presence or absence of of 2OHOA or palmitate (150 µM). The cells were then washed twice with phosphate-buffered saline (PBS; 137 mM NaCl, 2.7 mM potassium chloride, 12 mM dibasic sodium phosphate, 1.38 mM monobasic potassium phosphate [pH 7.4]), resuspended in 500 µl of methanol and vortexed. The cells were subsequently incubated at 4°C for 1 h, and then for 30 min at room temperature with 100 µg/ml ethidium bromide and 100 µg/ml RNAse A (Sigma-Aldrich) in PBS. Single-cell ethidium bromide fluorescence (25,000 events) was measured on a Coulter Epics XL flow cytometer using EXPO 32 flow cytometry software (Beckman Coulter, Inc.) with the gates set to differentiate between G_0_/G_1_, S and G_2_/M phases.

### Quantitative Reverse Transcription-Polymerase Chain Reaction (qRT-PCR)

1321N1 cells were seeded in 6-well plates containing 2 ml of culture medium (5% FBS) per well at density of 1×10^4^ cells/cm^2^. After incubating overnight, the cells were treated with 2OHOA or palmitate (150 µM) for 24 or 48 h and the regulatory effects of 2OHOA on *CHOP*, *IRE1α, ATF4, ATF6* and s*XBP1* mRNA expression was assessed by Real-time quantitative PCR (RT-qPCR). Total RNA was extracted from 1321N1 cells using the RNeasy Mini kit in combination with the RNase-free DNase kit (Qiagen, Hilden, Germany). Reverse transcription of total RNA (1 µg) was carried out in a final volume of 20 µl, containing the following reagents (Roche, Mannheim, Germany): anchored-oligo(dT) primer (2.5 µM); random hexamer primer (60 µM); dNTP mix (dGTP, dCTP, dATP, and dTTP, each at 1 mM); reverse transcriptase reaction buffer (8 mM MgCl_2_); RNase inhibitor (20 U); reverse transcriptase (10 U), and RNase-free water. The reaction mixtures were then incubated at 65°C (5 min), 37°C (50 min), and 70°C (15 min), and the cDNA samples obtained were then stored at −20°C. For PCR amplification, primers were designed based on the *CHOP, IRE1α, ATF4, ATF6* and *XBP1* sequences obtained from GenBank: 5′-CCG CAG CAG GTG CAG G-3′ (*XBP1* spliced forward primer) and 5′-GAG TCA ATA CCG CCA GAA TCC A-3′ (*XBP1* spliced reverse primer); 5′-GCC AAA ATC AGA GCT GGA ACC T-3′ (*CHOP* forward primer) and 5′-ACA GTG TCC CGA AGG AGA AAG G-3′ (*CHOP* reverse primer); 5′-TGT ACC ATT GAG GGA GAG GC-3′ (*IRE1α* forward primer) and 5′-GAG ACC CTG CGC TAT CTG AC-3′ (*IRE1α* reverse primer); 5′-TTC CTG AGC AGC GAG GTG TTG-3′ (*ATF4* forward primer) and 5′-TCC AAT CTG TCC CGG AGA AGG-3′ (*ATF4* reverse primer); 5′-TGA CAA AGC CCT GAT GGT GCT A-3′ (*ATF6* forward primer) and 5′-TGT TCC AGA GCA CCC TGA AGA A-3′ (*ATF6* reverse primer). As an endogenous control, *β-actin* expression of was determined in 1321N1 cells using the following primers: 5′-GCG GGA AAT CGT GCG TGA CAT T-3′ (forward) and 5′-CTA CCT CAA CTT CCA TCA AAG CAC-3′ (reverse). RT-qPCR amplifications were carried out on a Step One v 2.0 thermal cycler (Applied Biosystems) using the SYBR^®^ *Premix Ex Taq*™ (Perfect Real Time, Takara) containing *TaKaRa Ex Taq*™ HS, dNTP's, Mg^2+^, and the SYBR^®^ Green I and ROX™ Reference Dye. Thermal cycling was preceded by an initial denaturation step at 95°C for 5 min. DNA amplification and fluorescence quantification was performed over 35 cycles, with a denaturation step at 95°C for 5 s, and a 34 s annealing and extension step at 60°C. The melting curve was determined by one denaturation step at 95°C for 5 s followed by an annealing step for 34 s (55°C). Fluorescence quantification was performed after each DNA extension step (60°C), and the data was analyzed using Step One v 2.0 software. The ratio between the expression of *CHOP*, *IRE1α*, *ATF6* or *XBP1* and that of *β-actin* (for 1321N1 cells whose expression is not modulated by 2OHOA), was determined as described by Pfaffl *et al*., 2005 [40]. The results were expressed as ddCt values (as a percentage) using the following formula: ddCt = E X^(Ctc-Ctx)^/E Bact^(Ctc-Ctx)^. Efficiency (E) = 10^(−1/m)^. (m) = slope of the graph formed by Ct values of mRNA vs the logarithm (log) of its concentration (ng/µl). This value was used to calculate the relative expression in 2OHOA or palmitate-treated cells with respect to untreated (control) cells. The PCR products were further characterized by melting curve analysis and agarose gel electrophoresis.

### Fluorescence Microscopy

1321N1, SF-767 and MRC-5 cells were seeded in 4-well (1.7 cm^2^) plates containing 750 µl of culture medium (5% FBS) per well at densities of 1×10^4^ cells/cm^2^ (glioma cells) and 3×10^4^ cells/cm^2^ (MRC-5), respectively. After incubating overnight to allow cell attachment, cells were treated with 2OHOA or palmitate (150 µM) for 48 h and they were then incubated for 1 h with LysoSensor Green DND-189 pH Indicator (2 µM, pH 4.5–6: Invitrogen/Molecular probes). During the last 5 minutes of this incubation, Hoechst (trihydrochloride trihydrate) stain (40 µg/ml, Invitrogen/Molecular probes) was added to each well. The cells were examined on a Nikon Eclipse TE2000-S Fluorescence microscope (400X) and the photomicrographs of the acidic vesicles were analyzed using Image J 1.38x software (Wayne Rasband, National Institutes of Health; rsb.info.nih.gov).

### Electron microscopy

Cells were seeded at 1×10^4^ cells/cm^2^ in a Lab-Tek chamber slides of 4 wells (Nalge Nunc International, Naperville, IL) and were fixed in 3.5% glutaraldehyde for 1 hour at 37°C. Cells were postfixed in 2% OsO4 for 1 h at room temperature and stained in 2% uranyl acetate in the dark for 2 h at 4°C. Finally, cells were rinsed in sodium phosphate buffer (0.1 M, pH 7.2), dehydrated in ethanol, and infiltrated overnight in Araldite (Durcupan, Fluka, Buchs SG, Switzerland). Following polymerization, embedded cultures were detached from the chamber slide and glued to Araldite blocks. Serial semi-thin (1.5 µm) sections were cut with a diamond knife in a Leica ultramicrotome Ultracut UC-6 (Leica, Heidelberg, Germany) and mounted onto slides and stained with 1% toluidine blue. Selected semi-thin sections were glued (Super Glue, Loctite) to araldite blocks and detached from the glass slide by repeated freezing (in liquid nitrogen) and thawing. Ultrathin (0.07 µm) sections were prepared with a diamond knife ultracut and stained with lead citrate. Finally, photomicrographs were obtained under a transmission electron microscope (FEI Tecnai G2 Spirit Biotwin) using a digital camera (Morada, Soft Imaging System, Olympus).

### Statistics

The results were expressed as the mean±SEM of at least three independent experiments, and the level of significance was set at P*<*0.05 (Student's *t*-test).
